# The extent, frequency and ecological functions of food wasting by parrots

**DOI:** 10.1038/s41598-019-51430-3

**Published:** 2019-10-24

**Authors:** Esther Sebastián-González, Fernando Hiraldo, Guillermo Blanco, Dailos Hernández-Brito, Pedro Romero-Vidal, Martina Carrete, Eduardo Gómez-Llanos, Erica C. Pacífico, José A. Díaz-Luque, Francisco V. Dénes, José L. Tella

**Affiliations:** 1 Departament of Applied Biology, Universidad Miguel Hernández, Elche, Alicante Spain; 2 Department of Conservation Biology, Doñana Biological Station CSIC, Américo Vespucio 26, 41092 Sevilla, Spain; 3 Department of Evolutionary Ecology, Museo Nacional de Ciencias Naturales CSIC, Madrid, Spain; 4 Department of Physical, Chemical and Natural Systems, Universidad Pablo de Olavide, Sevilla, Spain; 5 Foundation for the Research and Conservation of Parrots in Bolivia (FPCILB), Barrio Estación Argentina, Calle Fermín Rivero 3460, Santa Cruz de la Sierra, Bolivia; 6 University of Alberta, Department of Biological Sciences, Edmonton, Alberta Canada

**Keywords:** Behavioural ecology, Macroecology

## Abstract

Anecdotic citations of food wasting have been described for parrots, but we lack a comprehensive knowledge about the extent of this behaviour, and its ecological and evolutionary implications. Here, we combine experimental and observational approaches to evaluate the spatial, temporal, typological and taxonomic extent of food wasting by parrots, to identify the ecological and evolutionary factors driving food wasting, and to assess the incidence of two ecological functions derived from food wasting, such as food facilitation to other animal species and secondary seed dispersal. We found that food wasting is a widespread behaviour found in all the studied parrot species. However, the proportion of food wasted differed among species and throughout the year. Parrots wasted more food during the non-breeding season, when they relied on exotic plants and on unripe fruits or seeds. We also recorded 86 animal species feeding on the food wasted by parrots, 27 of which potentially acted as secondary seed dispersers. Overall, our study emphasizes the universality of food wasting among parrots, and the important implications that this behaviour may have for the species involved (i.e., the parrot, the plant, the other species feeding on wasted food), and for the functioning of the whole ecosystem.

## Introduction

The way species interact with each other may have overarching implications for community organization and ecosystem functioning^1–3^. For example, several studies have documented that mutualistic animal-plant interactions, such as seed dispersal or pollination, drive important coevolutionary forces shaping the structure of both animal and plant communities^4,5^. Likewise, animal species may use each other as indicators of habitat quality, affecting the final distribution of the species in the communities^6^. Therefore, it is important to identify how species interact with each other and to understand the mechanisms shaping these interactions.

An interesting understudied behaviour related to interacting frugivorous animals and fruit-producing plants is food wasting (hereafter, waste). Howe^7^ found that monkeys discarded almost two-thirds of the seeds they handled, while Bosch & Wedde^8^ described how some parrots ate fruits only partially, discarding the rest. Anecdotic citations of waste by parrots can also be found in other dietary studies, both in places where animal species are native^9–12^ or exotic^13^. However, we lack a comprehensive understanding the ecological and evolutionary implications of this behaviour, as well as its implications.

Food wasting is illogical under classical ecological theories of resource use, where optimal foragers move between alternative patches^14^ or diet items (e.g.^15^) to prevent the total depletion of a particular resource. Predators may also maintain prey consumption within sustainable limits to ensure the viability of prey species^16^. Then, why do animals waste food? Some authors propose that waste may be an accidental behaviour. Frugivorous and granivorous species may unintentionally drop fruits and seeds while handling them during the foraging process^17,18^, larger fruits being involuntarily dropped with a higher frequency than small ones^17^. Other unexplored factors that may affect accidental dropping are conspecific disturbance or the coevolutionary history of the animal and the plant species. Conspecific density may affect waste, as individuals foraging in larger groups may drop more food than those in small ones. Besides, animals may drop more fruits from exotic than from native plants because they have had shorter evolutionary times together to get used to them. However, frugivores may also deliberately drop low-quality fruits such as those parasitized^11^, unripe, or those with low energetic and nutritional content^19^. In these cases, if waste is not accidental, individuals may be able to adjust this behaviour according to food availability, as dropping food when it is abundant does not have a large energetic cost.

Despite the uncertainty about why parrots waste food, several studies have suggested that this behaviour may benefit other species^9,10,13^. For example, dropped fruits or seeds become available to ground dwelling animals that otherwise cannot benefit from them^9,10,13^. Besides eating them, these species can also act as secondary dispersers^9–13^, increasing the number of different dispersal modes for the plant involved and, thus, the chances of effective dispersal. However, we do not know to what extent wasted food is consumed or secondarily dispersed by other species^20^.

Parrots (Psittaciformes order) are an evolutionarily old and diversified animal group^21^ where waste has been anecdotally observed, but never quantified. Therefore, we combined experimental approaches with fieldwork conducted in five continents to understand the importance of this behaviour across parrot species, its drivers and the consequences for other species. Table 1 summarizes the hypothesis tested about waste by parrots, as well as the associated predictions. Briefly, we evaluate the spatial, temporal, typological and taxonomic extent of waste by parrots. If waste is an anecdotal behaviour, we expect to find it only in few parrot species. Otherwise, if waste is a widespread behaviour, we would find it in most parrot species, independently of their evolutionary history and their region of origin.

**Table 1:** Tested hypotheses, associated predictions, reasoning of the prediction, variable used to test the prediction, dataset used and verification of the prediction based on our analyses.

Hypothesis	Prediction	Rationale	Variable	Dataset used	Verified?
Waste is widespread	1. Waste happens in many species, all the year, on many plant parts, in all biogeographic regions		Presence of waste	Field transects Experiment	Yes Yes
	2. Waste is independent of the evolutionary history of the species		Phylogenetic signal	Experiment	Yes
	3. Waste may happen in large amounts		Amount of food wasted	Experiment Waste quantification	Yes Yes
Waste is not random	4. Waste occurrence and quantity differ among species	Species differ in their handling abilities and foraging strategies	Presence of waste Amount of food wasted	Field transects Experiment	Yes Yes
Waste is accidental	5. Waste is more frequent in large bird groups	Because of conspecific disturbance	Number individuals trial	Experiment	No
	6. Waste is more frequent with exotic plant species	Because parrots are less used to them	Origin plant	Field transects	Partially
Waste is deliberate	7. Amount of food wasted is larger in large species	Small species have fast metabolisms and need to optimize resources	Body mass	Experiment	No
	8. Waste is lower when food availability is reduced	To cover nutritional requirements	Fasting	Experiment	No
	9. Wasted food is less energetic (unripe)	Parrots select high-quality food	Ripening status	Field transects	Partially
	10. Waste is more frequent outside the breeding season	Because energetic requirements are higher during breeding	Season	Field transects	Yes
	11. Wasted food has more parasites	Parrots select high-quality food	Parasites presence	Waste quantification	No
Waste benefits other species	12. Waste is large under the tree	Attracts animals	Num. fruit/seed under tree	Waste under tree	Yes
	13. Wasted food is used by a variety of species	Because it is a good alternative resource	Species detected	Camera traps Direct observations	Yes Yes
	14. A number of benefited species may also act as secondary dispersers, with different dispersal distances		Detected species traits	Camera traps Direct observations	Yes Yes

Then, we assessed if waste is a random process, independent of parrot life traits. To so do, we looked for differences in waste occurrence and amount of food wasted among species to identify the ecological factors driving waste. We hypothesized that this behaviour can be either accidental (i.e. plant parts fall because of difficulties during handling) or deliberate (i.e. parrots decide to waste food). Accidental waste may happen with a higher frequency when individuals forage in large groups, because of conspecifics disturbance, or with exotic plants, because parrots are less used to handle them. Contrarily, if waste is deliberate, this behaviour should happen more frequently with low-quality fruits and seeds (e.g. parasitized and unripe), and when energetic requirements are softer (e.g. outside the reproductive season, or when food availability is high). We also predict that smaller species will waste less food because they have a faster metabolism than large species (e.g.^22^) and need to optimize food intake. Lastly, we hypothesized that wasted food can benefit other species, so we assessed the extent and incidence of two possible ecological functions derived from waste: food facilitation to other animal species and seed dispersal. If wasted food is beneficial for the plant and for other species, we expect that the number of seeds underneath the trees where parrots have wasted food will be large and will attract many species. We also expect that waste will benefit a large number of different species with different potential dispersal modes. Finally, we discuss some possible ecological functions of waste and their ecological and evolutionary consequences.

## Results

### Food wasting extent and randomness

**Figure 1:**
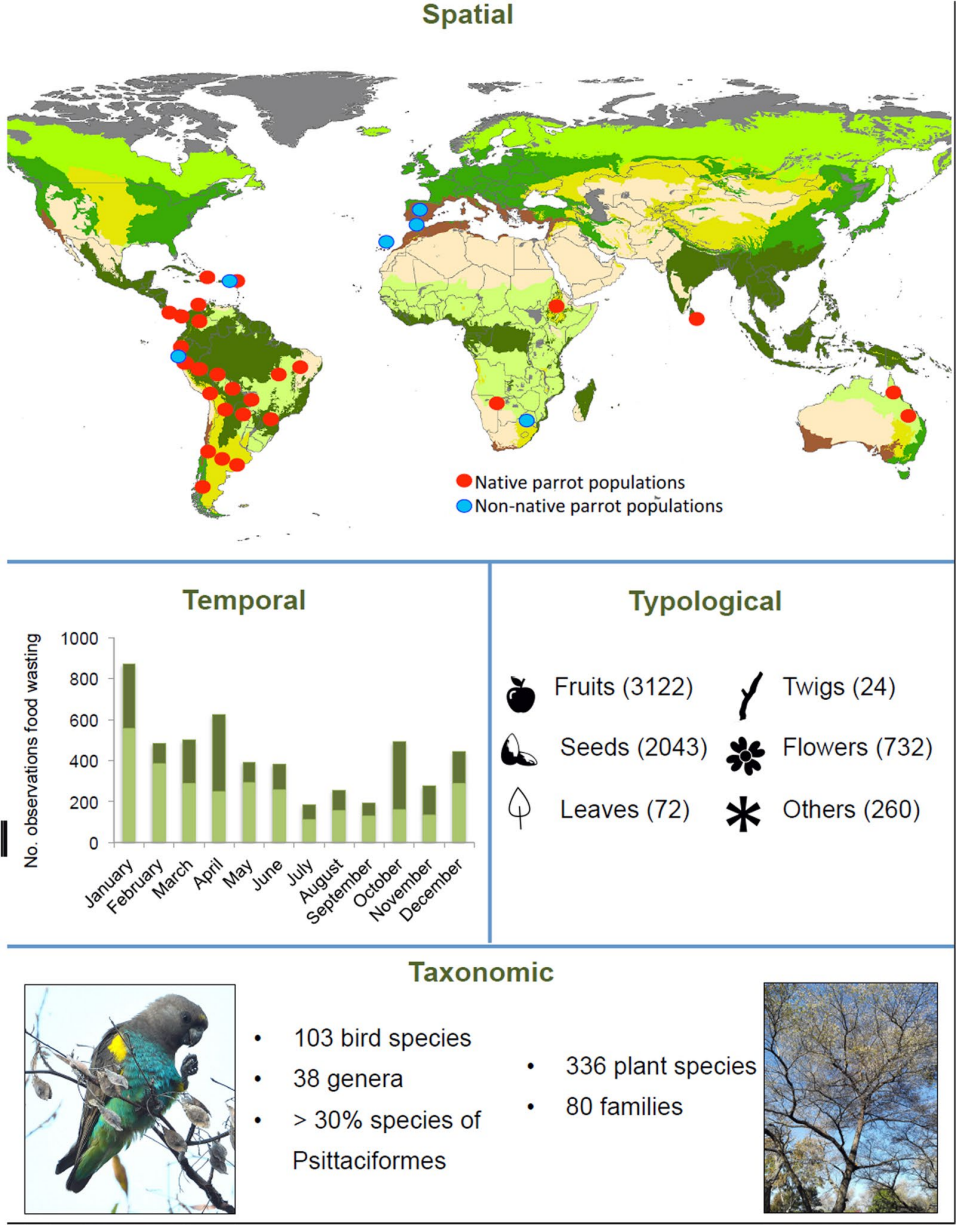
The spatial, temporal, typological and taxonomic extent of food wasting by parrots: Spatial: Each circle shows a surveyed area. Blue circles represent areas where parrots are exotic species, while red circles are areas included in their native distribution. Temporal: The graph shows the total number of waste observations per month by all parrot species (light green) and, specifically, by *Psittacula kramerii* (dark green). Typological: Number of observations for different plant parts wasted by parrots. Others include invertebrates, stems, sprouts, resin and tree bark. Taxonomic: Number of bird species found wasting food and plant species involved. Pictures: *Poicephalus meyerii* (left) wasting *Terminalia sericea* fruits and seeds (right), pictures by J.L. Tella. Icons authors: Georgiana Ionescu (fruit), Shawn Erdely (seed), Mansion@design (flower), Noël Rasendrason (twig), Myly (leave) and Lisa Staudinger (asterisk), all from thenounproject.com.

Waste by parrots is a widespread behaviour in many aspects (P1, Fig. 1). It was found in all the study sites visited (35 biomes, 17 countries and 5 continents), in native and introduced parrot ranges. Waste was observed throughout the year, in both the breeding and non-breeding season. Parrots wasted mainly fruits and seeds, but also flowers, leaves, twigs, stems, sprouts, parasites and bark. We observed 103 parrot species (40 species in the experimental approach, 75 in the field and 12 in both the experiment and the field) from 38 genera wasting food from 336 plant species belonging to 80 families (see Supplementary Tables S1 and S2 for complete lists of parrots wasting food and plants where waste occurred).

Following our expectations (P2), waste was independent of the evolutionary history of the species, as it did not show a significant phylogenetic signal using any of the 100 phylogenies used (mean *K*: 0.104, range: 0.007–0.165, all *P* > 0.409). Besides, we found that the percentage of waste by parrots was large (P3), as it averaged 21.2% (SD = 13.0) of the total food provided to individuals during an *in-captivity* experiment (range: 0.4–72.7%). In the wild, we found that parrots wasted 11.8% (SD = 25.1) of the fruits and 14.6% (SD = 20.3) of the seeds that they handled, ranging from lack of waste to a waste of all the fruits or up to 80% of the seeds.

**Figure 2:**
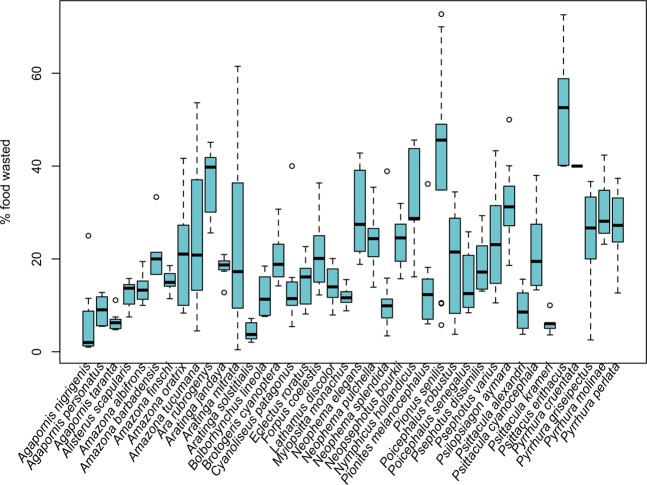
Boxplot of the percentage of food wasted by the 40 parrot species included in the experiment.

Moreover, we found strong among-species differences in waste occurrence in the wild (δAIC with null model 2882) and in the proportion of food wasted during the experiment (δAIC with null model 12, Fig. 2), suggesting that food wasting is not a random process (P4).

### Factors driving food wasting

**Figure 3:**
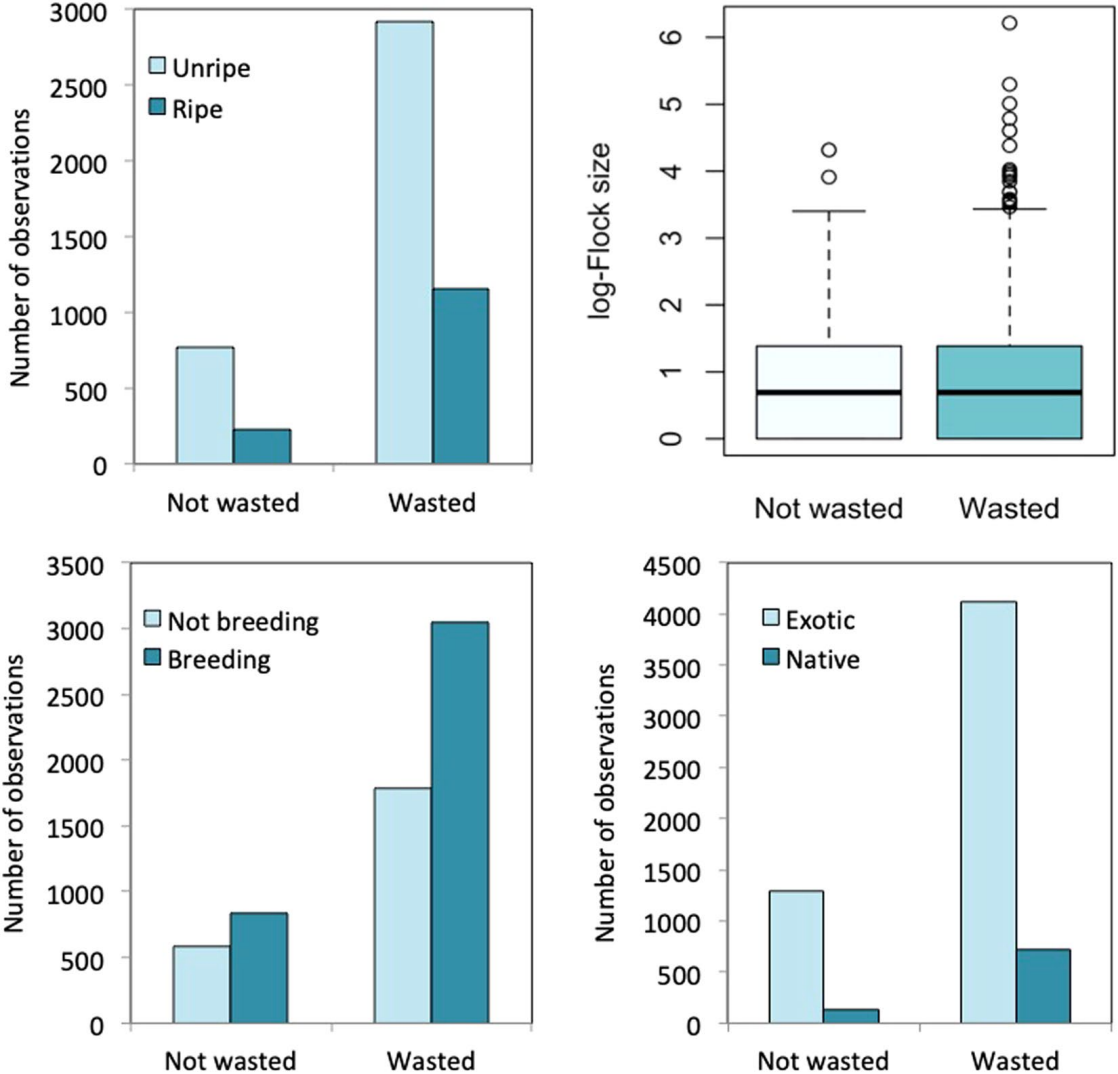
Representation of the food wasting occurrence (food wasted vs. food not wasted) in relation to the ripening stage of the fruit/seed (unripe/ripe), the log-flock size, the season when the observation was taken (breeding/non-breeding), and the origin of the plant (native/exotic) where the observation was taken.

Using a large database of field observations about waste occurrence we identified some factors affecting the occurrence of this behaviour. Fruit and seed wasting were more frequent during the non-breeding season (P10), in exotic plant species (P6) and on unripe fruits or seeds (P9) for all the species. We also repeated the analyses considering only data from *P. krameri*, which is the parrot species with more detailed information, and we observed the same pattern, both including all observations collected across the world and only those observed in Seville, the population with more information (Table 2, Fig. 3). This last analysis corroborates results obtained using the whole data. However, the analyses for all species but excluding *P. krameri* indicate that the season was the only factor driving waste occurrence.

**Table 2:** Models relating food wasting frequency (1/0) in foraging flocks with the number of individuals in the foraging flock (flock size), the season when the observation was taken (breeding/non-breeding), the ripening stage of the fruit/seed (green/ripe) and the origin of the plant (native/exotic) where the observation was made.

	All species	*P. krameri*	*P. krameri* (S)	Excluding *P. krameri*
N	4716	3141	1278	1575
Intercept	1.898	1.125	−0.593	2.398
Log (flock size)	0.358***	0.424***	0.703***	0.207*
Season: Breeding	−0.384***	−0.369***	−0.949***	−0.392*
Status: Green	0.455***	0.599***	0.820***	0.149
Plant: native	−0.298**	−0.372**	−0.983***	−0.199

The model for all species and for all species except *P. krameri* included the bird species as a random term. We show the number of observations used in each model (N), the coefficients of each variable. P-values as follows: ***P < 0.001, **P < 0.01, *P < 0.05.

Results obtained using experimental data showed that species size (i.e. average body mass, P7), food availability (i.e. fasting, P8) and conspecifics disturbance (i.e. number of individuals in the cage, P5) did not affect the proportion of food wasted (all p-values > 0.14, Supplementary Table S3). Finally, we also found that the total proportion of wasted fruits that were parasitized was very low (<4%, n = 176, P11).

### Ecological functions of food wasting

By checking the amount of food wasted under the trees where parrots had been wasting food, we found that this was large in many cases, as expected (P12). We found an average (±SD) of 53.4 ± 52.2 wasted fruits and 42.0 ± 54.9 wasted seeds under the trees (Supplementary Table S4). The maximum number of seeds found under a single tree (239) was larger than the number of fruits (164).

The number of species that benefited from the food wasted by parrots was also large and widely distributed, supporting P13, including 86 species of birds, mammals, reptiles, fishes, and ants (Table 3) that widely ranged in body size (0.002–750 kg). Also, we detected 28 different species from 26 genera potentially acting as secondary dispersers of the fruits and seeds wasted, including ants, birds, mammals and reptiles. As predicted (P14), these species ranged from very small to very large body sizes, and their average dispersal distances were mainly large (>30 m), but also very large (>100 m) for two species.

**Table 3:** Number of species detected consuming or as secondary dispersers of the fruits wasted by parrots by taxonomy, functional group, body size and dispersal distance.

		Food facilitation	Seed dispersal
Taxonomy	Species	86	27
	Genera	67	25
	Families	51	17
Group	Ants*	3	3
	Birds	53	13
	Mammals	27	9
	Reptiles	2	2
	Fishes	1	0
Body size	Very large (>10 kg)	13	2
	Large (>1 kg)	16	4
	Medium (>0.1 kg)	29	12
	Small (>0.01 kg)	23	6
	Very small (<= 0.01 kg)	5	3
Dispersal distance	Very large (>100 m)	—	2
	Large (>30 m)	—	11
	Medium (>10 m)	—	9
	Small (<= 10 m)	—	5

*This is a conservative number of species due to the hard species identification.

## Discussion

In this paper, we show that waste is not an anecdotic behaviour among parrots, but a widespread habit present in at least the 103 studied species. Waste is mainly focused on fruits and seeds, although it also involves other plant parts, and occurs throughout the year and through all the regions where parrots have native or introduced populations. We also show that waste has implications for other species that can forage or secondarily disperse wasted seeds and fruits. Thus, we can consider that parrots are interacting with plants along mutualism-antagonism continuums, as they can both prey upon several plant parts but also act as seed-dispersers, pollinators or plant healers^12,23^.

Besides waste being a widespread behaviour in parrots, we found that the proportion of food wasted changed between species, suggesting that some ecological or evolutionary factor may be modulating this behaviour. However, our tests to disentangle the accidental or deliberate nature of waste were not conclusive. We found that parrots waste more food from non-native plant species than from native ones, maybe because they had a shorter co-evolutionary history to adjust their handling techniques to the fruits and seeds of these novel species. Also, exotic plant species are often located closer to human infrastructures and thus, waste may be a consequence of human disturbance (i.e. parrots suddenly flying away and dropping food when humans get close to them). We also found that waste is more frequent during the non-breeding than during the breeding season, which is probably related to the higher nutritional requirements that individuals have while raising chicks^24^. If individuals need to gather more food for themselves and their chicks during the same time, they may choose to reduce food waste to increase foraging efficiency, suggesting that waste may also be deliberate. Also, parrots seem to waste with a higher frequency low quality (i.e. unripe) fruits, showing some type of selectivity. However, we did not find differences in food waste in relation to species body size or food availability (i.e. fasting), suggesting that metabolic differences among species and food availability are not driving this behaviour. Finally, some studies had suggested that parrots might preferentially waste or consume parasitized fruits, modulating the effect of parasites on plant fitness^11,25^. However, our observations did not show any preferential consumption of parasitized fruits.

Food wasted by parrots benefits many animal species, as seeds and fruits become more readily available and during a longer temporal window after being thrown from the tree by parrots. Also, wasted food is less often rotten or dry, as happens when food naturally falls from the trees. Parrots waste plenty of half-eaten fruits, reducing the original size of the fruit and breaking hard fruit parts, thus allowing their consumption by smaller species. For example, Douglas *et al*.^26^ found that the quantity of parrot frugivory increased habitat quality for Bananaquits (*Coereba flaveola*), because of parrot ability to open hard fruits and leave them partially consumed and thus available for other species. Besides, fruits become available for ground-dwelling species that otherwise cannot access the resource (as already suggested in^9,10,13^).

Fruits and seeds wasted by parrots can also be subject to secondary dispersal by many animals. This may be vital for some plant species, as many plants rely on secondary dispersal in their reproductive cycle^27,28^. It is important to underline that even if many of the wasted fruits and seeds are unripe and partially consumed, a large proportion of them can ripen and germinate after dispersal^20,29,30^. Also, having seed dispersers with different ecological characteristics (e.g. home ranges, places to defecate, dispersing methods) may increase the chances of effective seed dispersal^28^. The list of species either consuming food discarded by parrots or acting as secondary dispersers is very large, and includes many species from different taxonomic groups and with very different body sizes and dispersal distances. It is worth mentioning that our list is very conservative, as many of the species that we observed consuming the wasted fruits and seeds can also potentially disperse them. Thus, the impact of this wasteful behaviour on a large list of animal and plant species may be large given its universality.

Besides food facilitation and secondary seed dispersal, waste may have other ecological functions. If this behaviour has been maintained over the evolutionarily history of this old and diversified animal group^21^, it may be because it has some beneficial consequences for parrots^12^. One suggestion is that waste may benefit parrots by increasing the availability of high quality (e.g. large-sized, sugar-rich and nutrient-rich) fruits. In horticulture, it is widely known that such large high-quality fruits can be obtained through fruit and flower pruning^31,32^. Interestingly, pruning needs to be directed to unripe fruits to affect the quality of the non-wasted fruits^33–35^, coinciding with our result that waste occurs with a higher frequency in unripe fruits. Moreover, fruit and flower pruning are also known to reduce gaps between fructifications^36^ or reduce biennial bearing^32,37,38^. Thus, parrots may also be extending the fruiting period of the trees and increasing their predictability (i.e. shorter gaps between fructifications and transition from biennial to annual bearing). For any of these hypotheses to be possible, parrots need to be able to make intertemporal choices (i.e. sacrifice short-term satisfaction to obtain a higher reward in the future), which have already been detected for this bird group^39^. Overall, waste may have longer-time effects on wasted plants than expected, but further studies are needed to validate this hypothesis.

Besides the apparent negative effect that fruit and flower wasting may have for the plant, it is widely known that many plant species naturally produce more flowers and fruits than they can set^36^. The overproduction of juvenile fruits may be evolutionarily adaptive as these can satiate pre-dispersal fruit and seed predators^40,41^ and plants may be able to produce larger yields than average in occasional years of plentiful resources^42,43^. Plants may also compensate this overproduction by aborting flowers or fruits during the growth process^36^. As wasted fruits are preferentially unripe, their loss can be partially compensated by reducing fruit or flower abortion, reducing the negative effect of fruit thinning for the plant.

Waste may also have important consequences for the soil, as large amounts of organic matter accumulate over short time periods under a single tree. This behaviour may also expedite nutrient cycling, as wasted plant parts may enter the decomposition phase faster than if they were to stay in the tree for longer time periods^12^. Overall, our study wants to emphasize the universality of waste in parrots and the important implications this behaviour may have for the species involved (i.e. plants and parrots), but also show how other species benefit from the wasted food and for the functioning of the ecosystem.

Two specifications about our study need to be done before concluding. First, it is important to notice that the experimental data cannot be directly compared with what happens in the natural environment as the food provided and the way parrots obtain is markedly different. However, the waste experiment was designed to control for several factors that would be very challenging to account for in the wild. For example, it is very difficult to test among-species differences on the wild because the species are never found under the same circumstances (e.g., environmental conditions, food species). Another important factor that cannot be tested in the field but was easily addressed in the experiment was food availability. Therefore, the experiment offers complementary information to that provided by the data gathered in the field. Also, in our predictions 7–11, we assume that an individual wasting less food has a higher foraging efficiency than an individual wasting more food. However, it may be more efficient to feed by partially eating and wasting various fruits than to handle the same fruit for a long time, which will become smaller and more difficult to eat. This does not invalidate our predictions, as about half of the fruits and seeds wasted are intact (see Supplementary Table S3).

Despite the several aspects that deserve more research, this study adds to the growing evidence that parrots have a much more important role for the conservation of the ecosystems than previously thought^23,44^. Some of the functions attributed to parrots, such as primary and secondary dispersal [^12,45^, this study], may have a very important role in the current scenario of global change where tropical forests are being fragmented at high rates and forest recovery may depend on the efficiency of seed dispersal. However, many parrot species are highly endangered, with several species functionally extinct and many of the remaining species under strong human pressure (e.g., 28% of extant species are classified as threatened under IUCN^46^). Thus, management efforts to conserve parrot species should considerate the ecological interactions of food waste in the conservation planning strategies of habitat protection and population recovery (e.g. selection of priority areas for conservation and selection of release sites).

## Methods

### Datasets used

To test our hypotheses about waste, we combined information gathered using different approaches and unified in five different datasets (All of them available as Supplementary Material).

#### Dataset 1

Experiment in captivity. We first quantified waste experimentally using captive individuals. We performed 362 experimental trials using 130 individuals from 40 parrot species belonging to 24 genera (body weight range: 33–550 g) to measure the proportion of food wasted by the species. Individuals were kept in groups of 1–4 individuals, simulating their typical aggregated foraging behaviour. As cages used for the experiment always have the same size, experiments done including a larger number of individuals simulate situations of higher conspecific disturbance. Parrots were acclimated to the cages and diet for 10 days prior to the experiment. In a third of the experiments, individuals were fasted 24 hours before the start of the experiment, to simulate low food availability. Each species was fed with the typical food used in captivity (e.g. mixtures of millet, birdseed, sunflowers, and peanuts). Food was weighted (±0.1 g) and provided ad libitum to the parrots. Cages were set so that wasted food could not be eaten by individuals after falling to the cage floor. Each group of birds was tested approximately five times, and each trial lasted 24 hours. Wasted food was weighted, including only those seeds that were intact (i.e. half consumed seeds were not included). Finally, we calculated the proportion of food wasted by each group of parrots in each trial. All experimental protocols are in accordance with the relevant guidelines and regulations. Birds were kept in captivity under permit SGYB/FOA/AFR from the Consejería de Medio Ambiente, Junta de Andalucía, in the authorized centre for experimental avian research SE/16/U (REGA ES410910008016).

#### Dataset 2

Field transects. We collected observations of waste in different fieldwork campaigns performed in 17 countries and 5 continents (Fig. 1a). We looked for groups of foraging parrots in pre-defined roadside and walking transects (see details about transects on^47^). Every time we detected a foraging group, we observed focal parrot groups or individuals for 5–10 minutes and annotated the occurrence of food wasting (1/0), the parrot species, the plant species consumed, the part of the plant that was wasted (flowers, fruits, seeds, bark, leaves, twigs, sprouts, stems, resin or invertebrates), flock size, the season when the observation was taken (breeding/non-breeding), the ripening stage of the fruits/seeds (unripe/ripe), date, site and the origin of the plant where the observation was taken (native/exotic). This dataset includes a total of 6253 observations of foraging parrots observed between 2011 and 2019 in 37,612 km of transects.

#### Dataset 3

Waste quantification. We quantified the proportion of food wasted in the field by observing the foraging behaviour of individuals detected handling fruits or seeds during the field transects. For this and the following datasets, and for the statistical analyses in this study, we focused on fruits or seeds and excluded other wasted plant parts because fruits and seeds are the main food types wasted by parrots (see Fig. 1). Foraging individuals were observed from a distance with binoculars or telescopes. We identified the bird and plant species, and we counted the flock size and the number of fruits/seeds each individual ate or wasted. We then calculated the proportion of food wasted as the number of wasted fruits/seeds divided by the total number of fruits/seeds handled. We compiled 412 observations of individual birds from 20 species in Bolivia, Costa Rica, Namibia, Brazil, Peru, Argentina and Spain, between 2014 and 2019. Data was collected during six different months in both the breeding and non-breeding season, and birds handled 1841 fruits and 934 seeds. As some studies suggest that parrots may be wasting parasitized fruits or seeds (e.g.^11^), we also counted the total number of wasted fruits with worms for 176 fruits under 7 different tree species during fieldwork in the Brazilian *cerrado* in 2017.

#### Dataset 4

Waste under tree. We estimated the number of fruits/seeds a group of parrots could waste per individual tree. To do so, we counted the total number of intact and wholly or partially eaten fruits/seeds under a tree after a group of parrots foraged on it. We also identified the plant species. When the number of fruits was very large or the area was hard to screen because of the dense vegetation, we counted half of the area under the tree and then doubled the number of fruits/seeds. We compiled information on 98 trees from 29 species in Australia, Peru, Ecuador, Bolivia and Brazil between 2013 and 2017.

#### Dataset 5

Camera traps and direct observations. We used 96 camera traps to monitor the animal species using fruits and seeds wasted by parrots. Cameras were located under the plant, in front of a bunch of fruits/seeds. They stayed activated 5–7 days during 24 hours. Data was gathered in Brazil and Bolivia, under four different plant species where waste had been observed: *Attalea totai*, *A. barreirensis*, *A. speciosa* and *Mauritia flexuosa*. From the pictures, we separated species that consumed the fruits/seeds and those that took entire fruits/seeds out of the camera, thus being possible secondary seed dispersions. We combined this information with 293 direct observations of food facilitation and secondary dispersal taken in Australia, Spain, Puerto Rico, South Africa, Argentina and Sri Lanka between 2012 and 2019.

### Food wasting extent

To test our first prediction (P1) and describe the spatial extent of waste, we identified all the areas around the world where we had observed waste in the wild. The temporal extent of waste was described as the total number of waste events recorded per month. We also calculated the total number of waste events for ring-necked parakeets *Psittacula krameri*, the species with the largest number of waste events detected. For the temporal extent we used the *Field transects* dataset, as this is the largest compilation of waste events taken using a standardized method. This same dataset was used to identify the typological extent of waste by counting the total number of waste events found for each plant type (flower, fruit, seed, bark, leaves, twigs, sprouts, stems or invertebrates). Finally, the taxonomic extent of waste by parrots was quantified by identifying the total number of parrot species that was found wasting food and the total number of plant species that were subject to waste by parrots in any of our datasets, and in non-systematic observations performed during fieldwork.

Our second prediction (P2) that waste is independent of the evolutionary history of the species was explored using data from the *Experiment*. We assessed if there was a phylogenetic signal in the proportion of food experimentally wasted by the different species using the descriptive statistics *K*^48^. When *K* < 1, the relatives resemble each other less than expected under the Brownian motion evolution, while when *K* > 1 close relatives are more similar than expected under the Brownian evolution. We evaluated the statistical significance of the phylogenetic signal by comparing the observed variance of independent contrasts of the proportion of food experimentally wasted by the different parrot species to a null model of shuffling taxa labels across the tips of the phylogeny. We calculated *K* and the statistical significance of the phylogenetic signal for 100 bird phylogenies from Jetz *et al*.^49^ using the *picante* package^50^ in R version 3.5.3^51^.

Finally, we evaluated the proportion of food wasted (P3) using two datasets: the *Experiment* and the *Waste quantification*. We used the average (±SD) proportion of food wasted by each group of parrots in each trial for the *Experiment* data and the average (±SD) number of wasted fruits/seeds by each individual parrot for the *Waste quantification* data.

### Waste differences among species

If food wasting is a not a random process (P4) waste occurrence and quantity should differ among species. We used the *Field transects* data to compare waste occurrence (1/0) among species and the *Experiment* dataset to compare the proportion of food wasted among species. To test if waste occurrence differed between species, we fitted a Generalized Linear Model (GLM) in R with species as a predictor variable and occurrence as a response variable, using a binomial distribution. We then compared the proportion of food wasted (response variable) among species (predictor variable) in the experiment by means of Generalized Linear Mixed Models (GLMM) using a beta distribution with the *glmmADMB* library^52^. Because the same individuals were used for different trials, we included individual (or group of individuals) as a random factor in all the models. In both cases, we compared the model including species as predictor variable with a null one. Models with a difference in AIC smaller than 10 were considered equally supported.

### Factors driving food wasting

Waste may be driven by different factors, depending on its accidental or deliberate behaviour (predictions 5–11, Table 1). Because the different factors affecting waste may be related, we fitted multivariate models including several predictor variables at the same time. We ran one model for waste occurrence (1/0) (*Field transects* dataset) and one for the proportion of wasted food (*Experiment* dataset). *Experiment* data were used to relate the number of individuals in the cage during the trial (P5), parrot body size (mean weight in g, P7) and the reduction in food availability (simulated by a fasting period, P8) (predictor variables) with the proportion of food wasted (dependent variable) by means of GLMMs using a beta distribution. We included individual (or group of individuals) as a random factor in all the models, nested within species. The weight of the birds was standardized before modelling (i.e. transformed to have a mean of 0 and standard deviation of 1).

Then, we evaluated factors affecting waste occurrence (1/0) in relation with the season when the observation was taken (breeding/non-breeding, P10), the ripening stage of the fruit/seed (unripe/ripe, P9) and the origin of the plant where the observation was taken (native/exotic, P6). We also included the number of individuals in the flock as a covariate in the models to control for the potential effect of larger flocks having a higher chance of showing waste. To test the consistency of the results, we performed the analyses for all the species, but also for the species with the largest number of observations (*P. krameri*); additionally, only for *P. krameri* in the study site with a larger number of observations (Seville, Spain, where the species is introduced) and finally, for all species excluding *P. krameri*. We fitted Generalized Linear Models (GLM) in R using a binomial error distribution for the models for *P. krameri* and GLMMs with species as a random term for the model with all the species and for the model with all the species, but excluding *P. krameri*.

As some studies suggest that parrots may be wasting parasitized fruits or seeds (P11), we calculated the proportion of wasted fruits or seeds that were parasitized for 176 fruits found underneath seven different tree species.

### Ecological functions of food wasting

To test if waste is large under the tree (P12), we used the *Waste under tree* dataset. We calculated the mean (±SD) number of intact and partially eaten fruits/seeds under a tree. We also identified the maximum number of intact and partially eaten fruits and seeds found.

We finally evaluated two possible ecological functions of waste by parrots, facilitation of food to other species and secondary seed dispersal (P13 & P14), using the *Camera traps* *and direct observations* dataset. For each species benefiting from wasted food, we identified its taxonomic group (i.e. ant, bird, mammal, reptile or fish) and estimated its body size (i.e. very large [>10 kg], large [>1 kg], medium [>0.1 kg], small [>0.01 kg], very small [<= 0.01 kg]) using published studies (see reference list in Dataset). For those species detected acting as secondary dispersers, we also identified the mean dispersal distance from the literature (i.e. very large [>100 m], large [>30 m], medium [>10 m], small [<= 10 m]).

## Supplementary information

Supplementary Tables

Dataset 1

Dataset 2

Dataset 3

Dataset 4

Dataset 5

## Data Availability

All data generated or analysed during this study are included in this published article (and its Supplementary Information Files).
