# Supplementary material for: The extent, frequency and ecological functions of food wasting by parrots
Source: Sci Rep. 2019 Oct 24;9:15280. doi: 10.1038/s41598-019-51430-3 (PMC6813306; doi:10.1038/s41598-019-51430-3)
Supplement: Supplementary file 1 — Supplementary Tables [file 41598_2019_51430_MOESM1_ESM.docx]

**The extent, frequency and ecological functions of food wasting by parrots**

E. Sebastián-González, F. Hiraldo, G. Blanco, D. Hernández-Brito, P. Romero-Vidal, M. Carrete, E. Gómez-Llanos, E.C. Pacífico, J.A. Díaz-Luque, F.V. Denés, J.L. Tella

**Supplementary Material**

**Table S1.** List of bird species found wasting food. We show if the species was observed wasting food in the experimental approach, in an area where the species is native or in an area where the species is exotic. We also show the region of origin of the species.

| **Species** | **Region** | **Experiment** | **Exotic** | **Native** |
| --- | --- | --- | --- | --- |
| *Agapornis nigrigenis* | Afrotropical | 1 | 0 | 0 |
| *Agapornis personatus* | Afrotropical | 1 | 1 | 0 |
| *Agapornis roseicollis* | Afrotropical | 0 | 1 | 0 |
| *Agapornis taranta* | Afrotropical | 1 | 0 | 1 |
| *Alipiopsitta xanthops* | Neotropical | 0 | 0 | 1 |
| *Alisterus scapularis* | Australasia | 1 | 0 | 0 |
| *Amazona aestiva* | Neotropical | 0 | 1 | 1 |
| *Amazona albifrons* | Neotropical | 1 | 0 | 1 |
| *Amazona amazonica* | Neotropical | 0 | 1 | 1 |
| *Amazona auropalliata* | Neotropical | 0 | 0 | 1 |
| *Amazona autumnalis* | Neotropical | 0 | 0 | 1 |
| *Amazona barbadensis* | Neotropical | 1 | 0 | 0 |
| *Amazona finschi* | Neotropical | 1 | 0 | 0 |
| *Amazona guatemalae* | Neotropical | 0 | 0 | 1 |
| *Amazona lilacina* | Neotropical | 0 | 0 | 1 |
| *Amazona ochrocephala* | Neotropical | 0 | 1 | 1 |
| *Amazona oratrix* | Neotropical | 1 | 0 | 0 |
| *Amazona pretrei* | Neotropical | 0 | 0 | 1 |
| *Amazona tucumana* | Neotropical | 1 | 0 | 0 |
| *Amazona ventralis* | Neotropical | 0 | 0 | 1 |
| *Amazona vinacea* | Neotropical | 0 | 0 | 1 |
| *Amazona vitatta* | Neotropical | 0 | 0 | 1 |
| *Anodorhynchus hyacinthinus* | Neotropical | 0 | 0 | 1 |
| *Anodorhynchus leari* | Neotropical | 0 | 0 | 1 |
| *Ara ambiguus* | Neotropical | 0 | 0 | 1 |
| *Ara ararauna* | Neotropical | 0 | 1 | 1 |
| *Ara chloropterus* | Neotropical | 0 | 0 | 1 |
| *Ara macao* | Neotropical | 0 | 0 | 1 |
| *Ara militaris* | Neotropical | 0 | 0 | 1 |
| *Ara rubrogenys* | Neotropical | 1 | 0 | 1 |
| *Ara severus* | Neotropical | 0 | 0 | 1 |
| *Aratinga jandaya* | Neotropical | 1 | 0 | 1 |
| *Aratinga nenday* | Neotropical | 0 | 1 | 0 |
| *Aratinga solstitialis* | Neotropical | 1 | 0 | 0 |
| *Bolborhynchus lineola* | Neotropical | 1 | 0 | 0 |
| *Brotogeris chiriri* | Neotropical | 0 | 0 | 1 |
| *Brotogeris cyanoptera* | Neotropical | 1 | 0 | 0 |
| *Brotogeris jugularis* | Neotropical | 0 | 0 | 1 |
| *Brotogeris pyrrhoptera* | Neotropical | 0 | 0 | 1 |
| *Brotogeris versicolorus* | Neotropical | 0 | 1 | 1 |
| *Cacatua alba* | Indomalaya | 0 | 1 | 0 |
| *Cacatua galerita* | Australasia | 0 | 0 | 1 |
| *Cacatua moluccensis* | Indomalaya | 0 | 1 | 0 |
| *Calyptorhynchus banksii* | Australasia | 0 | 0 | 1 |
| *Calyptorhynchus funereus* | Australasia | 0 | 0 | 1 |
| *Cyanoliseus patagonus* | Neotropical | 1 | 0 | 1 |
| *Diopsittaca cumanensis* | Neotropical | 0 | 0 | 1 |
| *Diopsittaca nobilis* | Neotropical | 0 | 0 | 1 |
| *Eclectus roratus* | Australasia | 1 | 0 | 0 |
| *Enicognathus ferrugineus* | Neotropical | 0 | 0 | 1 |
| *Enicognathus leptorhynchus* | Neotropical | 0 | 0 | 1 |
| *Eupsittula aurea* | Neotropical | 0 | 0 | 1 |
| *Eupsittula cactorum* | Neotropical | 0 | 0 | 1 |
| *Eupsittula canicularis* | Neotropical | 0 | 1 | 1 |
| *Eupsittula nana* | Neotropical | 0 | 0 | 1 |
| *Eupsittula pertinax* | Neotropical | 0 | 0 | 1 |
| *Forpus coelestis* | Neotropical | 1 | 0 | 0 |
| *Lathamus discolor* | Australasia | 1 | 0 | 0 |
| *Melopsittacus undulatus* | Australasia | 0 | 1 | 0 |
| *Myiopsitta luchsi* | Neotropical | 0 | 0 | 1 |
| *Myiopsitta monachus* | Neotropical | 1 | 1 | 1 |
| *Neophema elegans* | Australasia | 1 | 0 | 0 |
| *Neophema pulchella* | Australasia | 1 | 0 | 0 |
| *Neophema splendida* | Australasia | 1 | 0 | 0 |
| *Neopsephotus bourkii* | Australasia | 1 | 0 | 0 |
| *Nymphicus hollandicus* | Australasia | 1 | 0 | 0 |
| *Ognorhynchus icterotis* | Neotropical | 0 | 0 | 1 |
| *Orthopsittaca manilata* | Neotropical | 0 | 0 | 1 |
| *Pionites melanocephalus* | Neotropical | 1 | 0 | 0 |
| *Pionus maximiliani* | Neotropical | 0 | 1 | 1 |
| *Pionus menstruus* | Neotropical | 0 | 0 | 1 |
| *Pionus senilis* | Neotropical | 1 | 0 | 1 |
| *Poicephalus meyeri* | Afrotropical | 0 | 0 | 1 |
| *Poicephalus robustus* | Afrotropical | 1 | 0 | 0 |
| *Poicephalus senegalus* | Afrotropical | 1 | 1 | 0 |
| *Polytelis swainsonii* | Australasia | 0 | 1 | 0 |
| *Primolius auricollis* | Neotropical | 0 | 0 | 1 |
| *Primolius couloni* | Neotropical | 0 | 0 | 1 |
| *Psephotus dissimilis* | Australasia | 1 | 0 | 0 |
| *Psephotus varius* | Australasia | 1 | 0 | 0 |
| *Psilopsiagon aymara* | Neotropical | 1 | 0 | 0 |
| *Psittacara chloropterus* | Neotropical | 0 | 0 | 1 |
| *Psittacara erythrogenys* | Neotropical | 0 | 1 | 1 |
| *Psittacara finschi* | Neotropical | 0 | 0 | 1 |
| *Psittacara frontata* | Neotropical | 0 | 0 | 1 |
| *Psittacara hockingi* | Neotropical | 0 | 0 | 1 |
| *Psittacara leucophtalmus* | Neotropical | 0 | 0 | 1 |
| *Psittacara mitratus* | Neotropical | 1 | 0 | 1 |
| *Psittacula alexandri* | Indomalaya | 1 | 0 | 0 |
| *Psittacula calthrapae* | Indomalaya | 0 | 0 | 1 |
| *Psittacula cyanocephala* | Indomalaya | 1 | 0 | 0 |
| *Psittacula eupatria* | Indomalaya | 0 | 0 | 1 |
| *Psittacula krameri* | Indomalaya | 1 | 1 | 1 |
| *Psittacara wagleri* | Neotropical | 0 | 0 | 1 |
| *Psittacus erithacus* | Afrotropical | 1 | 0 | 0 |
| *Pyrilia haematotis* | Neotropical | 0 | 0 | 1 |
| *Pyrrhura cruentata* | Neotropical | 1 | 0 | 0 |
| *Pyrrhura frontalis* | Neotropical | 0 | 0 | 1 |
| *Pyrrhura griseipectus* | Neotropical | 1 | 0 | 0 |
| *Pyrrhura molinae* | Neotropical | 1 | 0 | 1 |
| *Pyrrhura perlata* | Neotropical | 1 | 0 | 0 |
| *Thectocercus acuticaudatus* | Neotropical | 0 | 1 | 1 |
| *Trichoglossus haematodus* | Australasia | 0 | 0 | 1 |

**Table S2.** List of plant species where parrots wasted food. We show the species, family and the number of times we detected food wasting at the species.

| **Species** | **Family** | **Number of observations** |
| --- | --- | --- |
| *Acacia angustissima* | Fabaceae | 16 |
| *Acacia aroma* | Fabaceae | 1 |
| *Acacia auriculiformis* | Fabaceae | 12 |
| *Acacia karroo* | Fabaceae | 13 |
| *Acacia mearnsii* | Fabaceae | 10 |
| *Acacia melanoxylon* | Fabaceae | 11 |
| *Acaena splendens* | Rosaceae | 1 |
| *Acrocomia totai* | Arecaceae | 65 |
| *Aesculus hippocastanum* | Sapindaceae | 1 |
| *Agonandra brasiliensis* | Opiliaceae | 2 |
| *Ailanthus altissima* | Simaroubaceae | 2 |
| *Aiphanes minima* | Arecaceae | 2 |
| *Aizoon canariense* | Aizoaceae | 1 |
| *Albizia carbonara* | Fabaceae | 1 |
| *Albizia guachapele* | Fabaceae | 2 |
| *Albizia julibrissin* | Fabaceae | 1 |
| *Albizia procera* | Fabaceae | 9 |
| *Albizia saman* | Fabaceae | 19 |
| *Anacardium excelsum* | Anacardiaceae | 3 |
| *Anacardium occidentale* | Anacardiaceae | 9 |
| *Anadenathera colubrina* | Fabaceae | 2 |
| *Anisocapparis speciosa* | Capparidaceae | 4 |
| *Annona muricata* | Annonaceae | 2 |
| *Arachis hypogaea* | Fabaceae | 36 |
| *Araucaria angustifolia* | Araucariaceae | 303 |
| *Araucaria araucana* | Araucariaceae | 34 |
| *Araucaria bidwillii* | Araucariaceae | 51 |
| *Araucaria cunninghamii* | Araucariaceae | 5 |
| *Arbutus unedo* | Ericaceae | 7 |
| *Ardisia escallonioides* | Primulaceae | 4 |
| *Asparagus arborescens* | Asparagaceae | 2 |
| *Aspidosperma quebracho-blanco* | Apocynaceae | 2 |
| *Astronium urundeuva* | Anacardiaceae | 1 |
| *Attalea butyracea* | Arecaceae | 2 |
| *Attalea phalerata* | Arecaceae | 0 |
| *Attalea princeps* | Arecaceae | 3 |
| *Attalea speciosa* | Arecaceae | 9 |
| *Attalea totai* | Arecaceae | 1 |
| *Averrhoa carambola* | Oxalidaceae | 3 |
| *Azadirachta indica* | Meliaceae | 2 |
| *Bactris gasipaes* | Arecaceae | 1 |
| *Bambusa vulgaris* | Poaceae | 4 |
| *Betula pendula* | Betulaceae | 4 |
| *Brachychiton populneus* | Malvaceae | 3 |
| *Brosimum alicastrum* | Moraceae | 1 |
| *Bucida buceras* | Combretaceae | 4 |
| *Bursera simaruba* | Burseraceae | 3 |
| *Bursera tomentosa* | Burseraceae | 2 |
| *Byrsonima crassifolia* | Malpighiaceae | 7 |
| *Byrsonima pachyphylla* | Malpighiaceae | 34 |
| *Byrsonima verbascifolia* | Malpighiaceae | 5 |
| *Cajanus cajan* | Fabaceae | 1 |
| *Callicarpa ampla* | Lamiaceae | 3 |
| *Calophyllum antillarum* | Calophyllaceae | 6 |
| *Carapa guianensis* | Meliaceae | 2 |
| *Carduus thoermeri* | Asteraceae | 112 |
| *Carya illinoinensis* | Juglandaceae | 7 |
| *Cassia javanica* | Fabaceae | 2 |
| *Castanea sativa* | Fagaceae | 3 |
| *Castilla elastica* | Moraceae | 1 |
| *Casuarina cristata* | Casuarinaceae | 1 |
| *Casuarina equisetifolia* | Casuarinaceae | 11 |
| *Catalpa bignonioides* | Bignoniaceae | 7 |
| *Cecropia peltata* | Urticaceae | 1 |
| *Cecropia schreberiana* | Urticaceae | 9 |
| *Cedrela odorata* | Meliaceae | 15 |
| *Cedrus atlantica* | Pinaceae | 1 |
| *Cedrus libani* | Pinaceae | 1 |
| *Ceiba pentandra* | Malvaceae | 28 |
| *Ceiba penthandra* | Malvaceae | 1 |
| *Ceiba speciosa* | Malvaceae | 9 |
| *Celtis australis* | Ulmaceae | 45 |
| *Centaurea solstitialis* | Asteraceae | 46 |
| *Cerastium glomeratum* | Caryophyllaceae | 3 |
| *Ceratonia siliqua* | Fabaceae | 5 |
| *Cercis siliquastrum* | Fabaceae | 67 |
| *Chenopodium murale* | Chenopodiaceae | 4 |
| *Cipadessa baccifera* | Meliaceae | 1 |
| *Citharexylum donnell-smithii* | Verbenaceae | 1 |
| *Citharexylum spinosum* | Verbenaceae | 7 |
| *Citrus × aurantium* | Rutaceae | 2 |
| *Citrus × sinensis* | Rutaceae | 37 |
| *Citrus aurantifolia* | Rutaceae | 1 |
| *Clusia pratensis* | Clusiaceae | 1 |
| *Cnidoscolus quercifolius* | Euphorbiaceae | 1 |
| *Coccoloba caracasana* | Polygonaceae | 1 |
| *Coccoloba uvifera* | Polygonaceae | 52 |
| *Cocos nucifera* | Arecaceae | 7 |
| *Coffea arabica* | Rubiaceae | 5 |
| *Commiphora leptophloeos* | Burseraceae | 1 |
| *Compsoneura sprucei* | Myristicaceae | 1 |
| *Condalia buxifolia* | Rhamnaceae | 8 |
| *Cordia alba* | Boraginaceae | 1 |
| *Cordia collococca* | Boraginaceae | 5 |
| *Cordia cymosa* | Boraginaceae | 1 |
| *Corymbia clarksoniana* | Myrtaceae | 7 |
| *Corymbia tessallaris* | Myrtaceae | 2 |
| *Couepia grandiflora* | Chrysobalanaceae | 15 |
| *Couepia polyandra* | Chrysobalanaceae | 1 |
| *Couma macrocarpa* | Apocynaceae | 2 |
| *Crataegus monogina* | Rosaceae | 1 |
| *Croton magdalenensis* | Euphorbiaceae | 1 |
| *Cupania americana* | Sapindaceae | 3 |
| *Cupressus sempervirens* | Cupressaceae | 87 |
| *Curatella americana* | Dilleniaceae | 16 |
| *Cyanophalla retusa* | Capparidaceae | 2 |
| *Cyperaceus sp.* | Cyperaceae | 1 |
| *Cyttaria harioti* | Cyttariaceae | 2 |
| *Delonix regia* | Fabaceae | 15 |
| *Dendropanax latilobus* | Araliaceae | 1 |
| *Dovyalis caffra* | Salicaceae | 47 |
| *Dracaena draco* | Asparagaceae | 2 |
| *Drimys winteri* | Winteraceae | 1 |
| *Elaeis guianensis* | Arecaceae | 1 |
| *Emmotum nitens* | Icacinaeae | 7 |
| *Eriobotrya japonica* | Rosaceae | 75 |
| *Erythrina caffra* | Fabaceae | 21 |
| *Erythrina fusca* | Fabaceae | 16 |
| *Erythrina poeppigiana* | Fabaceae | 10 |
| *Erythroxylum deciduum* | Erythroxylaceae | 6 |
| *Eucalyptus camaldulensis* | Myrtaceae | 45 |
| *Eucalyptus globulus* | Myrtaceae | 35 |
| *Eucalyptus miniata* | Myrtaceae | 5 |
| *Eucryphia cordifolia* | Cunoniaceae | 1 |
| *Eugenia uniflora* | Myrtaceae | 38 |
| *Ficus benghalensis* | Moraceae | 19 |
| *Ficus carica* | Moraceae | 115 |
| *Ficus insipida* | Moraceae | 2 |
| *Ficus lyrata* | Moraceae | 27 |
| *Ficus macrophylla* | Moraceae | 57 |
| *Ficus maxima* | Moraceae | 1 |
| *Ficus microcarpa* | Moraceae | 106 |
| *Ficus nymphaeifolia* | Moraceae | 1 |
| *Ficus religiosa* | Moraceae | 9 |
| *Ficus rubiginosa* | Moraceae | 16 |
| *Ficus sp.* | Moraceae | 1 |
| *Ficus stuhlmannii* | Moraceae | 6 |
| *Ficus trigonata* | Moraceae | 2 |
| *Flacourtia inermis* | Salicaceae | 5 |
| *Fraxinus angustigolia* | Oleaceae | 33 |
| *Geoffroea decorticans* | Fabaceae | 1 |
| *Gleditsia triacanthos* | Fabaceae | 18 |
| *Gliricidia sepium* | Fabaceae | 9 |
| *Gmelina arborea* | Lamiaceae | 15 |
| *Grevillea glauca* | Proteaceae | 3 |
| *Grevillea robusta* | Proteaceae | 91 |
| *Guarea guidonia* | Meliaceae | 27 |
| *Guazuma ulmifolia* | Malvaceae | 1 |
| *Hancornia speciosa* | Apocynaceae | 3 |
| *Handroantus chrysanthus* | Bignoniaceae | 2 |
| *Helianthus annuus* | Asteraceae | 99 |
| *Heliocarpus sp.* | Malvaceae | 1 |
| *Hibiscus rosa-sinensis* | Malvaceae | 4 |
| *Hirtella rugosa* | Chrysobalanaceae | 2 |
| *Hymenaea courbaril* | Fabaceae | 1 |
| *Hymenaea stigonocarpa* | Fabaceae | 17 |
| *Hypochaeris radicata* | Asteraceae | 2 |
| *Inga adenophylla* | Fabaceae | 1 |
| *Inga chocoensis* | Fabaceae | 1 |
| *Inga cylindrica* | Fabaceae | 3 |
| *Inga edulis* | Fabaceae | 1 |
| *Inga feuilleei* | Fabaceae | 1 |
| *Inga punctata* | Fabaceae | 1 |
| *Inga spectabilis* | Fabaceae | 11 |
| *Jacaranda mimosifolia* | Bignoniaceae | 39 |
| *Jatropha hieronymi* | Euphorbiaceae | 11 |
| *Jatropha humboldtiana* | Euphorbiaceae | 7 |
| *Jatropha mollisima* | Euphorbiaceae | 4 |
| *Juglans regia* | Juglandaceae | 3 |
| *Juniperus procera* | Cupressaceae | 1 |
| *Juniperus virginiana* | Cupressaceae | 9 |
| *Lagerstroemia indica* | Lythraceae | 2 |
| *Lagerstroemia speciosa* | Lythraceae | 9 |
| *Laureliopsis philippiana* | Atherospermataceae | 2 |
| *Leucaena leucocephala* | Fabaceae | 2 |
| *Ligustrum japonicum* | Oleaceae | 38 |
| *Ligustrum lucidum* | Oleaceae | 13 |
| *Livistona chinensis* | Arecaceae | 165 |
| *Luehea seemannii* | Malvaceae | 4 |
| *Magonia pubescens* | Sapindaceae | 2 |
| *Malus domestica* | Rosaceae | 17 |
| *Mangifer indica* | Anacardiaceae | 96 |
| *Mangifera zeylanica* | Anacardiaceae | 1 |
| *Manilkara bidentata* | Sapotaceae | 3 |
| *Maprounea guianensis* | Euphorbiaceae | 2 |
| *Maranthes panamensis* | Chrysobalanaceae | 7 |
| *Mauritia flexuosa* | Arecaceae | 46 |
| *Mauritiella armata* | Arecaceae | 1 |
| *Maytenus boaria* | Celastraceae | 5 |
| *Melaleuca citrina* | Myrtaceae | 17 |
| *Melia azedarach* | Meliaceae | 311 |
| *Melicoccus bijugatus* | Sapindaceae | 10 |
| *Miconia impetiolaris* | Melastomataceae | 10 |
| *Misodendrum linearifolium* | Misodendraceae | 2 |
| *Morus alba* | Moraceae | 84 |
| *Morus nigra* | Moraceae | 127 |
| *Mouriri pusa* | Melastomataceae | 2 |
| *Musa × paradisiaca* | Poaceae | 3 |
| *Myrceugenia exsucca* | Myrtaceae | 1 |
| *Myrtus communis* | Myrtaceae | 16 |
| *Neea theifera* | Nyctaginaceae | 16 |
| *Nephelium lappaceum* | Sapindaceae | 1 |
| *Nothofagus dombeyi* | Nothofagaceae | 3 |
| *Nothofagus obliqua* | Nothofagaceae | 10 |
| *Ochroma pyramidale* | Malvaceae | 1 |
| *Olea europaea* | Oleaceae | 49 |
| *Olea europaea sylvestris* | Oleaceae | 21 |
| *Onopordum acanthium* | Asteraceae | 1 |
| *Oryza sativa* | Poaceae | 11 |
| *Pachira quinata* | Malvaceae | 10 |
| *Parkia biglandulosa* | Fabaceae | 4 |
| *Parkinsonia aculeata* | Fabaceae | 3 |
| *Parkinsonia praecox* | Fabaceae | 1 |
| *Parodiodendron marginivillosum* | Picrodendraceae | 2 |
| *Passiflora edulis* | Passifloraceae | 2 |
| *Patellifolia patellaris* | Amaranthaceae | 5 |
| *Patellifolia procumbens* | Amaranthaceae | 1 |
| *Peltophorum pterocarpum* | Fabaceae | 1 |
| *Persea americana* | Lauraceae | 11 |
| *Phalaris canariensis* | Poaceae | 3 |
| *Phoenix canariensis* | Arecaceae | 260 |
| *Phoenix dactylifera* | Arecaceae | 437 |
| *Phytolacca dioica* | Phytolaccaceae | 33 |
| *Pinus pinea* | Pinaceae | 13 |
| *Pinus radiata* | Pinaceae | 7 |
| *Pithecellobium dulce* | Fabaceae | 9 |
| *Plantago lanceolata* | Plantaginaceae | 2 |
| *Platanus × hispanica* | Platanaceae | 68 |
| *Platanus orientalis* | Platanaceae | 1 |
| *Platimiscium curuense* | Fabaceae | 1 |
| *Platypodium elegans* | Fabaceae | 1 |
| *Plocama pendula* | Rubiaceae | 5 |
| *Populus alba* | Salicaceae | 45 |
| *Populus deltoides* | Salicaceae | 33 |
| *Populus nigra* | Salicaceae | 31 |
| *Populus tremuloides* | Salicaceae | 3 |
| *Pouteria ramiflora* | Sapotaceae | 31 |
| *Prestoea montana* | Arecaceae | 6 |
| *Prosopis alba* | Fabaceae | 15 |
| *Prosopis caldeana* | Fabaceae | 2 |
| *Prosopis kuntzei* | Fabaceae | 4 |
| *Prunus avium* | Rosaceae | 47 |
| *Prunus cerasifera* | Rosaceae | 35 |
| *Prunus cerasus* | Rosaceae | 18 |
| *Prunus domestica* | Rosaceae | 6 |
| *Prunus dulcis* | Rosaceae | 135 |
| *Prunus malus* | Rosaceae | 1 |
| *Prunus persica* | Rosaceae | 17 |
| *Prunus salicina* | Rosaceae | 6 |
| *Pseudobombax septenatum* | Malvaceae | 6 |
| *Pseudosamanea guachapele* | Fabaceae | 1 |
| *Psidium cattleianum* | Myrtaceae | 1 |
| *Psidium guajava* | Myrtaceae | 26 |
| *Pterodon emarginatus* | Fabaceae | 1 |
| *Punica granatum* | Lythraceae | 16 |
| *Pyrus bourgaeana* | Rosaceae | 11 |
| *Pyrus communis* | Rosaceae | 25 |
| *Qualea amoena* | Vochysiaceae | 3 |
| *Quercus ilex* | Fagaceae | 3 |
| *Rhaphithamnus spinosus* | Verbenaceae | 1 |
| *Rheedia achachairu* | Clusiaceae | 2 |
| *Ribes magellanicus* | Grossulariaceae | 1 |
| *Richeria grandis* | Phyllanthaceae | 24 |
| *Robinia pseudoacacia* | Fabaceae | 35 |
| *Roystonea borinquena* | Arecaceae | 60 |
| *Sabal domingensis* | Arecaceae | 1 |
| *Salix alba* | Salicaceae | 1 |
| *Salix babylonica* | Salicaceae | 3 |
| *Salix caprea* | Salicaceae | 2 |
| *Salvertia convallariodora* | Vochysiaceae | 1 |
| *Samanea saman* | Fabaceae | 1 |
| *Sapium saltense* | Euphorbiaceae | 2 |
| *Saribus rotundifolius* | Arecaceae | 7 |
| *Schefflera actinophylla* | Araliaceae | 1 |
| *Schinopsis lorentzii* | Anacardiaceae | 21 |
| *Schinopsis marginata* | Anacardiaceae | 1 |
| *Schinus molle* | Anacardiaceae | 77 |
| *Schinus sp.* | Anacardiaceae | 1 |
| *Schinus terebinthifolius* | Anacardiaceae | 37 |
| *Sclerolobium aureum* | Fabaceae | 3 |
| *Senegalia gilliesii* | Fabaceae | 1 |
| *Senna siamea* | Fabaceae | 8 |
| *Serjania polyphylla* | Sapindaceae | 3 |
| *Silybum marianum* | Asteraceae | 52 |
| *Simarouba amara* | Simaroubaceae | 27 |
| *Sonchus tenerrimus* | Asteraceae | 3 |
| *Spathodea campanulata* | Bignoniaceae | 148 |
| *Spondias dulcis* | Anacardiaceae | 9 |
| *Spondias mombin* | Anacardiaceae | 1 |
| *Spondias purpurea* | Anacardiaceae | 8 |
| *Sterculia apetala* | Malvaceae | 7 |
| *Sterculia balanghas* | Malvaceae | 4 |
| *Sterculia foetida* | Malvaceae | 4 |
| *Styphnolobium japonicum* | Fabaceae | 58 |
| *Swartzia jorori* | Fabaceae | 1 |
| *Syagrus coronatus* | Arecaceae | 5 |
| *Syagrus romanzoffiana* | Arecaceae | 7 |
| *Syzygium malaccense* | Myrtaceae | 5 |
| *Syzygium smithii* | Myrtaceae | 8 |
| *Tabebuia rosea* | Bignoniaceae | 4 |
| *Tamarindus indica* | Fabaceae | 138 |
| *Tamarix gallica* | Tamaricaceae | 5 |
| *Tapirira guianensis* | Anacardiaceae | 3 |
| *Tapirira obtusa* | Anacardiaceae | 1 |
| *Taraxacum officinale* | Asteraceae | 3 |
| *Tebebuia impetiginosa* | Bignoniaceae | 1 |
| *Tectona grandis* | Verbenaceae | 5 |
| *Terminalia bucidoides* | Combretaceae | 2 |
| *Terminalia catappa* | Combretaceae | 87 |
| *Terminalia muelleri* | Combretaceae | 3 |
| *Terminalia sericea* | Combretaceae | 6 |
| *Terminalia sericea* | Combretaceae | 1 |
| *Thespesia grandiflora* | Malvaceae | 8 |
| *Thespesia populnea* | Malvaceae | 1 |
| *Thevetia peruviana* | Apocynaceae | 1 |
| *Tipuana tipu* | Fabaceae | 17 |
| *Toona ciliata* | Meliaceae | 5 |
| *Trachycarpus fortunei* | Arecaceae | 3 |
| *Trifolium repens* | Fabaceae | 1 |
| *Ulmus minor* | Ulmaceae | 29 |
| *Vallesia glabra* | Apocynaceae | 1 |
| *Vassobia breviflora* | Solanaceae | 1 |
| *Vitex cymosa* | Lamiaceae | 0 |
| *Vitex gigantea* | Lamiaceae | 1 |
| *Vochysia divergens* | Vochysiaceae | 1 |
| *Vochysia ferruginea* | Vochysiaceae | 3 |
| *Vochysia pilosa* | Vochysiaceae | 1 |
| *Vochysia tucanorum* | Vochysiaceae | 2 |
| *Washingtonia filifera* | Arecaceae | 147 |
| *Washingtonia robusta* | Arecaceae | 87 |
| *Ximenia americana* | Olacaceae | 1 |
| *Zea mays* | Poaceae | 12 |
| *Ziziphus joazeiro* | Rhamnaceae | 2 |
| *Ziziphus mauritiana* | Rhamnaceae | 1 |
| *Ziziphus mistol* | Rhamnaceae | 6 |
| *Zygia latifolia* | Fabaceae | 0 |

**Table S3.** Results of the model relating the proportion of food wasted in the experiment in relation to the average body mass of the parrot species, the number of individuals in the cage during the experiment and the presence of a fasting period before the trial. We show the model coefficient of the variable and the p-value.

| **Variable** | **Coefficient** | **P-value** |
| --- | --- | --- |
| Weight | -0.1195 | 0.14 |
| Fasting presence | -0.0989 | 0.16 |
| Number of individuals | -0.0397 | 0.78 |

**Table S4.** Summary of the number of fruits/seeds found under a tree after a group of parrots foraged on it. We include information on the total number of trees counted, mean (± SD) and maximum number of fruits/seeds found behind the trees, and mean (± SD) and maximum number of intact and half-eaten fruits/seeds.

|  | **Fruits** | **Seeds** |
| --- | --- | --- |
| Number trees counted | 57 | 72 |
| Number fruits/seeds per tree | 53.4 ± 53.2 | 41.98 ± 54.9 |
| Intact | 23.28 ± 29.44 | 13.8 ± 27.53 |
| Maximum | 164 | 130 |
| Half-eaten | 27.5 ± 25.7 | 11.4 ± 33.6 |
| Maximum | 92 | 239 |
